# Stepped wedge cluster randomized controlled trial designs: a review of reporting quality and design features

**DOI:** 10.1186/s13063-017-1783-0

**Published:** 2017-01-21

**Authors:** Michael J. Grayling, James M. S. Wason, Adrian P. Mander

**Affiliations:** MRC Biostatistics Unit Hub for Trials Methodology Research, Cambridge Institute of Public Health, University Forvie Site, Robinson Way, Cambridge, CB2 0SR UK

**Keywords:** Cluster randomized controlled trial, Reporting quality, Review, Stepped wedge

## Abstract

**Background:**

The stepped wedge (SW) cluster randomized controlled trial (CRCT) design is being used with increasing frequency. However, there is limited published research on the quality of reporting of SW-CRCTs. We address this issue by conducting a literature review.

**Methods:**

Medline, Ovid, Web of Knowledge, the Cochrane Library, PsycINFO, the ISRCTN registry, and ClinicalTrials.gov were searched to identify investigations employing the SW-CRCT design up to February 2015. For each included completed study, information was extracted on a selection of criteria, based on the CONSORT extension to CRCTs, to assess the quality of reporting.

**Results:**

A total of 123 studies were included in our review, of which 39 were completed trial reports. The standard of reporting of SW-CRCTs varied in quality. The percentage of trials reporting each criterion varied to as low as 15.4%, with a median of 66.7%.

**Conclusions:**

There is much room for improvement in the quality of reporting of SW-CRCTs. This is consistent with recent findings for CRCTs. A CONSORT extension for SW-CRCTs is warranted to standardize the reporting of SW-CRCTs.

**Electronic supplementary material:**

The online version of this article (doi:10.1186/s13063-017-1783-0) contains supplementary material, which is available to authorized users.

## Background

The randomized controlled trial (RCT) has long been considered the ‘gold standard’ for determining the effectiveness of an experimental intervention compared with a contemporary control [1]. Consequently, much research has been conducted to determine how to best design, analyze, and report such trials [1–4]. Since the 1980s, a subset of possible RCT designs has been given substantial attention; the cluster randomized controlled trial (CRCT) design [5]. As defined by Hayes and Moulton in 2009 [6], in a CRCT, ‘groups [are] randomly allocated to treatment arms. These groups are referred to as clusters […] Examples of clusters include schools, communities, factories, hospitals, or medical practices, but there are many other possible choices.’ In particular, such designs have been employed when it is inappropriate or impossible to use individual-level randomization, or when the group effects are of primary interest.

One possible CRCT design is the stepped wedge (SW); first proposed by Cook and Campbell [7], and first utilized in the Gambia Hepatitis Study [8]. In the SW-CRCT approach, the intervention is introduced over a number of time periods, with the order in which each cluster begins receiving the intervention determined at random. In the classical form of the design, all clusters begin in the control condition, and all receive the intervention by the completion of the trial, with all clusters actively taking part in the trial at each time period. Moreover, once a cluster switches to the intervention, it does not switch back to the control. However, whilst this latter feature remains common to all proposed SW-CRCT designs, recently numerous extensions have been proposed that, for example, allow some clusters to begin in the intervention, end in the control condition, or only actively take part in certain time periods [9].

In addition, there are several variations of the SW-CRCT according to the chosen method of data acquisition. For example, some trials may involve cross-sectional examination of a population, potentially accruing measurements from an entirely different set of subjects in each time period [10]. At the other end of the scale, they could involve gathering repeated measurements from a single cohort [11].

Indeed, much has been written in recent years about SW-CRCTs; from papers recommending how to design and analyze these trials, to reports on completed SW trials. Methodology [10–14] and software [15] now exists to determine required sample sizes and the optimal timings of the steps in a stepped wedge design have been ascertained [16, 17], whilst, as discussed, extensions to the standard design to allow for multiple levels of clustering or ‘incomplete block’ designs have also been presented [9]. Numerous papers have discussed when the design is more efficient, or preferential on alternate grounds, such as ethical or logistical grounds, to the parallel group CRCT [10, 18–27], whilst there have also been recommendations on how to analyze and report trials employing the SW-CRCT design [28]. Overviews of some of the benefits and limitations of SW-CRCTs can be found in the studies of de Hoop *et al.* [29], Hargreaves *et al.* [30], and Prost *et al.* [31].

However, little research has been conducted to determine the reporting quality of SW-CRCTs. The first two systematic reviews assessing SW-CRCTs [32, 33] were performed when few trials had used the design. Thus, they were unable to come to any strong conclusions about the quality of reporting; however, they did suggest that there was much room for improvement. Two more recently completed reviews [34] did give some consideration to the standard of reporting of completed SW-CRCTs. However, they only assessed a small selection of reporting quality indicators, primarily focusing on the employed analysis methods, and on trial reports published from 2010 to 2014. Similarly, Martin *et al.* [35] did assess reporting standards, but concentrated on nine indicators relating to sample size calculations. Finally, a 2016 review focused on the available statistical methodology for designing and analyzing SW-CRCTs that have been used to date [36]. Consequently, identification of any substantial reporting failures, unrelated to sample size calculation or trial analysis, remains an important step to be undertaken. Specifically, little is known about how well trials have been introduced, the results presented, or the trials discussed thereafter. In this work, we address this issue by conducting a literature review on SW-CRCTs with no limits on the calendar time, with 43 indicators of the quality of reporting considered, drawn from across the CONSORT statement for CRCTs [37, 38], allowing us to assess broadly the standard of reporting of conducted SW-CRCTs. In addition, we are able to provide a detailed analysis of numerous design features of SW-CRCTs.

## Methods

**Fig. 1:**
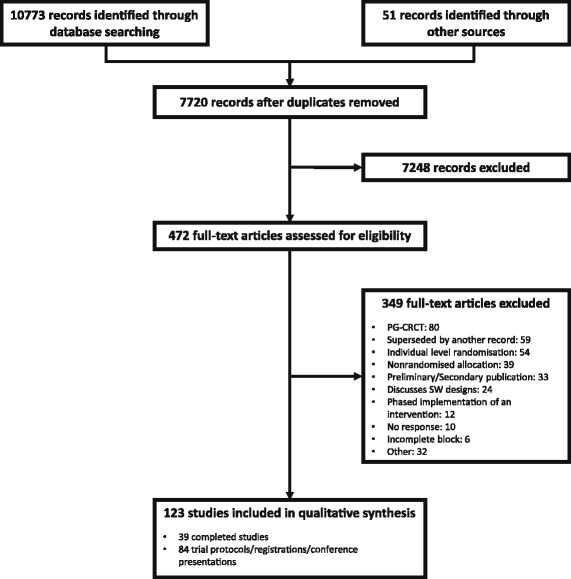
Flow diagram of records through the literature review. 123 studies were ultimately included in our review. PG-CRCT, parallel group clustered randomized controlled trial; SW, stepped wedge

A protocol of the literature review is provided as Additional file 1. A completed PRISMA checklist [39] is available as Additional file 2 and a flow diagram for the review appears in Fig. 1.

### Literature search strategy

PubMed (including Medline), Ovid (including Embase), Web of Knowledge, PsycINFO, the Cochrane Library, the ISRCTN registry and ClinicalTrials.gov were searched on 24 February 2015. No publication date restrictions were applied to the search. In addition, reference lists of the studies included in the final analysis were checked, along with those of several other relevant papers.

Based on the protocol of Mdege *et al.* [33], the following phrases were used: ‘stepped wedge’, ‘step wedge’, ‘experimentally staged introduction’, ‘delayed intervention’, ‘one directional crossover design’, and the 15 possible combinations of ‘incremental’/‘phased’/‘staggered’/‘step wise’/‘delayed’ and ‘recruitment’/‘introduction’/‘implementation’. Explicitly, these terms were entered for each database as follows:

- *Pubmed*. In the Advanced Search Builder, ‘All Fields’ was set to each of the 20 search terms in turn, e.g. ‘stepped wedge’. Results were downloaded as a.txt file.
- *Ovid*. Embase 1974 to 2015 Week 6 was included. In the Advanced Search tab, each of the 20 search terms were entered in turn as a keyword, e.g. ‘stepped wedge’. Results were downloaded as a.txt file.
- *PsycINFO*. In the Advanced Search tab, ‘Any Field’ was set to each of the 20 search terms in turn, e.g. ‘stepped wedge’. Results were downloaded as a.ris file.
- *Cochrane Library*. In the Search tab, ‘Search All Text’ was set to each of the 20 search terms in turn, e.g. ‘stepped wedge’. Results were downloaded as a.txt file.
- *Web of Knowledge*. In a Basic Search, ‘Topic’ was set to each of the 20 search terms in turn, e.g. ‘stepped wedge’. ‘TIMESPAN’ was set to ‘All years’. Results were downloaded as a.txt file.
- *ISRCTN registry*. In an Advanced Search, ‘Text search’ was set to each of the 20 search terms in turn, e.g. ‘stepped wedge’. Results were copied by hand (title of study, registry number, primary contact) into a.csv file.
- *ClinicalTrials.gov*. In an Advanced Search, ‘Search Terms’ was set to each of the 20 search terms in turn, e.g. ‘stepped wedge’. Results were downloaded as a.csv file.

Following this, identified records were merged into one.csv file for analysis using R v. 3.1.3 [40]. For further information, see Additional file 1.

### Study inclusion and exclusion criteria

Studies were included if they reported an original trial that used, or planned to use, a SW-CRCT design. Specifically, we here define a SW-CRCT to be any trial meeting the following criteria:

- Cluster-level allocation was utilized to compare experimental interventions with controls.
- Data were gathered in at least three time periods on at least two clusters.
- Each cluster begins in a control condition and changes from this control to an experimental intervention exactly once during the study, and then remains on this experimental intervention.
- The time point at which clusters change from control states to experimental ones is randomized, and not identical for all clusters.
- Data are gathered for every included cluster in every time period.
- An analysis is performed and reported on at least one primary outcome measure, for each experimental intervention, incorporating data from a time period in which all clusters received the intervention.

Note, therefore, that trials where clusters were not randomly allocated a time point for initiation of the experimental intervention were excluded, as were ‘before-and-after’ studies in which the time of switching to experimental interventions was equal for all clusters. Given our focus on CRCTs, trials where individual-level randomization was used were excluded. We also allow studies in which more than one experimental intervention is explored (being compared with identical or differing controls), but only if the clusters used to analyze each experimental intervention are entirely separate; we do not consider cases in which clusters switch between experimental interventions. Additionally, duplicate or secondary analysis publications for the same study were counted as one study, although data on the general characteristics of the design used (i.e. not the indicators of reporting standards) may have been extracted from several sources.

Further, we exclude trials in which some clusters were only offered an experimental intervention after the completion of the trial or data analysis, or where some clusters begin the trial on the experimental intervention. This is to focus on what many would consider the classical SW-CRCT design. It also ensures that parallel group CRCTs with baseline periods, or more than one follow-up period, are omitted without the need for additional inclusion criteria. Finally, our requirement that data must be gathered for every included cluster, in every time period, prevents the inclusion of ‘incomplete block’ SW-CRCTs [9]. Similarly, this allows us to concentrate on the reporting of the classical SW-CRCT design, and core features that should be present in this instance. It is probable that several additional reporting features should be present for more specialized SW-CRCT designs. Note, therefore, that in what follows, when we refer to SW-CRCTs, we mean what we have described here as the classical SW-CRCT, as prescribed by these criteria.

### Selection of studies for inclusion in the review

MJG screened the titles and abstracts of all records retrieved from the literature search to identify all potentially relevant full articles to be assessed for potential inclusion in the review. The full articles were then evaluated according to the pre-specified inclusion and exclusion criteria; being classified as included, excluded, or having insufficient information. For those with insufficient information to make a decision, the corresponding authors were contacted for further details (this, in particular, included studies where only abstracts or protocol summaries were available).

### Data extraction and analysis

Data were extracted from all included studies by MJG according to a pre-specified set of questions (see Additional file 1). The set included questions on study identifiers (such as lead author), the research area (such as disease or domain, setting and country), the motivations for using a SW-CRCT design, the general characteristics of the SW-CRCT design (such as number of clusters) and the data analysis method.

Additionally, for the included full trial reports, data were extracted on an additional 43 questions to assess the quality of reporting of SW-CRCTs (see Additional file 1 and Table 3). These criteria were selected according to those previously used [33], in addition to several based on the CONSORT extension to CRCTs [37, 38]. They were designed to assess the reporting quality of SW-CRCTs across all sections of the CONSORT statement. Whenever conclusions were unclear, final decisions were made through discussion with an additional author (JMSW or APM). The limitations of this record selection and data extraction procedure are discussed later in this article.

The reporting quality of the full trial reports was further examined by evaluating performance on the subset of the 43 assessed indicators listed on the CONSORT extensions to CRCTs; since the lack of an available CONSORT extension to SW-CRCTs makes this, at present, the best available advice for publication of SW-CRCTs, and it would therefore be hoped that trials would adhere to its guidance. Likewise, the quality of reporting was further graded on a subset of 10 of the 43 indicators chosen by the authors as key indicators. Additional file 1 provides further information on how these were chosen. Briefly, a group decision was made after discussions to choose the criteria that would (a) ease the identification of the trial as a SW-CRCT and justify its use, (b) allow the key design features of the utilized SW-CRCT to be determined and justified, and (c) determine that the primary results of the trial were soundly reported.

Moreover, as part of our review, data were also extracted, for each included full trial report, on whether a significant result was found for any of the trials primary endpoints. Within the literature, one of the primary reasons cited for using the SW-CRCT design is an inherent belief that the intervention will be effective; helping explain the desire to eventually implement it in all clusters. However, this may not always be the case.

We further categorize the data according to whether a minimum clinically important difference for the primary endpoints, for which a pre-specified power was to be achieved by the trial, was specified. That is, we assess whether a statistically significant result was ascertained on a primary endpoint for which a minimum clinically important difference had been provided. In this way, a single trial could then contribute to both categories (specified vs. unspecified minimum clinically important difference) if it evaluated performance on several primary endpoints, but only offered a minimum clinically important difference for a subset of these.

The final accrued data from our review were then analyzed, and figures produced, using R v. 3.3.2 [40].

## Results

### Literature search

The flow of the records through the review process is presented in Fig. 1. A total of 10,824 records were initially identified, of which 3104 were found to be duplicates. Title and abstract screening found 7248 to be irrelevant, leaving 472 for full article assessment to determine eligibility. Of these, 349 were found to be ineligible. This left 123 studies (39 full trial reports and 84 trial registrations, protocols or conference reports) meeting the eligibility criteria for inclusion in the review. A spreadsheet containing the extracted information on these studies is provided as Additional file 3. Note that these 123 studies include all of those from the previously conducted reviews [32–35, 41] that meet our chosen inclusion criteria.

### The where, when and why of SW-CRCTs

**Fig. 2:**
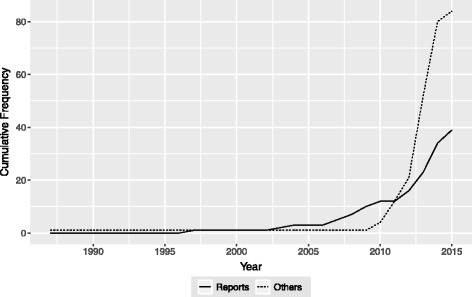
Use of the SW-CRCT design. The cumulative frequency of the designs use over time is displayed, according to the included reports and other records (trial registrations, protocols, conference reports)

Table 1 depicts the research areas in which SW-CRCT designs have been used, the countries in which the studies were carried out, whether industry support was received for the study, and the reasons given for utilization of a SW-CRCT. In addition, Fig. 2 displays the cumulative frequency of the publication year of each included study.

**Table 1:** Use of the SW-CRCT design (*N* = 123)

	Frequency (%)
Area	
Health	117 (95.1)
Education	3 (2.4)
Social	3 (2.4)
Country	
USA	20 (16.3)
UK	20 (16.3)
Netherlands	15 (12.2)
African	15 (12.2)
Australasian	12 (9.8)
Asian	10 (8.1)
Canada	9 (7.3)
South or Central American	8 (6.5)
Other European	6 (4.9)
France	5 (4.1)
Multiple	3 (2.4)
Received industry support	
Yes	6 (4.9)
No	108 (87.8)
Unclear	9 (7.3)
Stated reasons for use	
Everyone receives	51 (41.5)
None stated	45 (36.6)
Logistical or practical	43 (35.0)
Methodological	15 (12.2)
Time effects	13 (10.6)
Other	10 (8.1)
Cluster-level randomization	9 (7.3)
Prevent contamination bias	8 (6.5)
Encourage participation	7 (5.7)
Incorporate randomization	6 (4.9)
Efficiency grounds	6 (4.9)
Social or political acceptability	3 (2.4)
Fine-tune intervention	3 (2.4)

We observe that health-related research has overwhelmingly accounted for the majority of identified SW-CRCTs, with only a few examples of social and educational studies. This, however, may be a consequence of the databases used in our search. Nonetheless, within the health-related trials, an extremely broad selection of research domains can be observed, including, for example, HIV [42], asthma [43], hand hygiene [44], prostate cancer [45] and water contamination [46]. Moreover these trials have been conducted in a wide array of countries across Europe, North and South America, Asia and Africa, with the UK and USA accounting for the most, 20 studies each.

For a large majority of studies, no industry support was received (108/123, 87.8%). It can be seen that the design has been used substantially more often in recent years, with particularly notable spikes in trial registrations, protocols and conference presentations from 2010 to 2015.

Finally, the most common reason given for the use of the SW-CRCT design was a desire for the intervention to be made available to all clusters by the end of the trial, on ethical or equity grounds (51/123, 41.5%). The other commonly cited reasons included logistical or practical constraints (43/123, 35.0%), methodological considerations (relating, for example, to clusters acting as their own controls, having a comparison group at each time period, and the fact that it allows between- and within-cluster analyses) (15/123, 12.2%) and the ability to adjust for, or study the effect of, time (13/123, 10.6%). A large number of included studies did not state a reason for the designs use (45/123, 36.6%).

### Design characteristics of SW-CRCTs

**Fig. 3:**
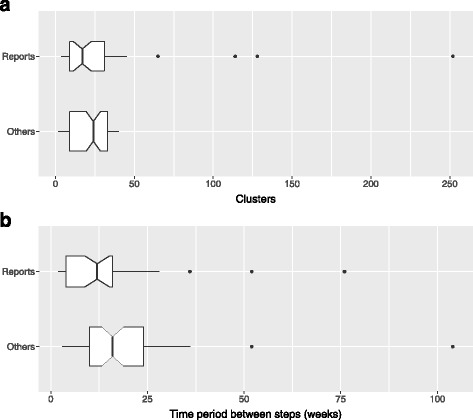
Design characteristics of SW-CRCTs. (**a**) Number of clusters and (**b**) time period between steps of the included studies (where clear) is depicted, according to whether the study record is either a completed report or other record type (trial registrations, protocols, conference reports). For completed reports, the final true figures are used; for the other records, the planned amounts are employed. For the box plot of the time periods between steps, 1 month was treated as 4 weeks and 1 year as 52 weeks. Notches are used to display the 95% confidence interval for the median, and the boxplot widths are proportional to the square root of the number of records on which they are based

Figure 3 and Table 2 together depict the general design characteristics of the included studies; the number of steps, number of clusters, time period between steps, the design type, and the number of participants (where each of these was obtainable) according to being a report (i.e. one of the 39 included completed studies) or other assessed record (i.e. one of the 84 included trial registrations, protocols or conference reports). Note that for the reports these figures are then the final attained values for the trial, whereas for the other records they are the planned values before trial commencement. Therefore, it should be remembered that the values for the other included records may not prove to be indicative of those ultimately used in SW-CRCTs.

**Table 2:** Design features of SW-CRCTs

	Reports (*N* = 39) (%)	Others (*N* = 84) (%)	All (*N* = 123) (%)
Design type			
Continuous recruitment short exposure	13 (33.3)	35 (41.7)	48 (39.0)
Open cohort	12 (30.8)	16 (19.0)	28 (22.8)
Closed cohort	12 (30.8)	17 (20.2)	29 (23.6)
Other	1 (2.6)	3 (3.6)	4 (3.3)
Unclear	1 (2.6)	13 (15.5)	14 (11.4)
Steps			
2	2 (5.1)	3 (3.6)	5 (4.1)
3	1 (2.6)	2 (2.4)	3 (2.4)
4	1 (2.6)	1 (1.2)	2 (1.6)
5	11 (28.2)	1 (1.2)	12 (9.8)
6	1 (2.6)	1 (1.2)	2 (1.6)
7	3 (7.7)	10 (11.9)	13 (10.6)
8	1 (2.6)	0 (0)	1 (0.8)
9	7 (17.9)	13 (15.5)	20 (16.3)
10	4 (10.3)	0 (0)	4 (3.3)
11	5 (12.8)	12 (14.3)	17 (13.8)
12	1 (2.6)	11 (13.1)	12 (9.8)
13	1 (2.6)	5 (6.0)	6 (4.9)
14	0 (0)	1 (1.2)	1 (0.8)
15	0 (0)	2 (2.4)	2 (1.6)
16	0 (0)	1 (1.2)	1 (0.8)
Unclear	1 (2.6)	21 (25.0)	22 (17.9)
Total number of participants	Reports (*N* = 36)	Others (*N* = 72)	All (*N* = 108)
Minimum	123.0	40.0	40.0
Q1	368.8	400.0	400.0
Median	1112.0	1200.0	1200.0
Q3	3264.8	6330.8	4000.0
Maximum	26456.0	256700.0	256700.0

We observe that SW-CRCTs have been, or are being, conducted with greatly different characteristics. The number of steps has varied from 2 to 16, with an overall median of 9, whilst the number of clusters has varied from 2 to 252, with an overall median value of 20.5. Furthermore, the median overall time period between steps was 12 weeks, but has varied from a few weeks up to 2 years. Finally, the total number of participants in SW-CRCTs has typically varied from a few hundred to a few thousand, with an overall median of 1200. However, there were instances of trials that planned to recruit only 40, or as many as 256,700, participants.

To date, the majority of SW-CRCTs have been of the continuous recruitment short exposure type (48/123, 39.0%) discussed for example in [41]; where, as the name suggests, participants are continually recruited and exposed for a short period before a measurement is made. There have, however, also been a large number of open (28/123, 22.8%) and closed cohort trials (29/123, 23.6%).

### Quality of reporting

**Fig. 4:**
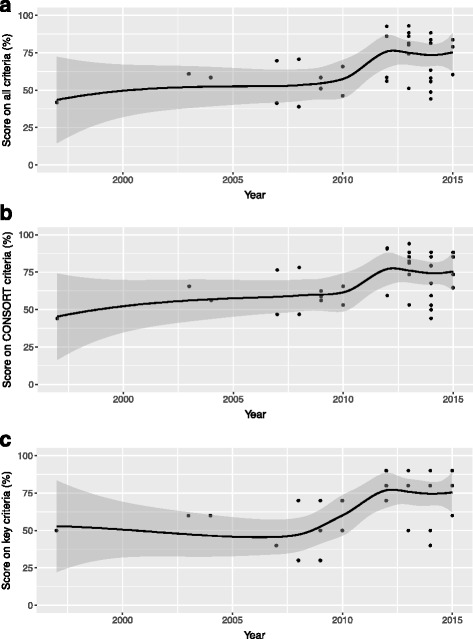
Quality of reporting over time. The percentage of the criteria that each study met against its publication year is depicted, according to (**a**) all considered criteria, (**b**) the subset listed on the CONSORT extension to CRCTs, and (**c**) the 10 criteria chosen as key. A Loess smoother is present on each graph to indicate time patterns, along with its 95% confidence interval

We assessed the quality of reporting of the 39 included full study reports using our 43 selected criteria. Summary results for each criteria are presented in Table 3. Details for each study can be found in Additional file 3. Additionally, Fig. 4 depicts a scatter graph of the percentage of the three sets of criteria met by each full report against publication year.

**Table 3:** Quality of reporting of SW-CRCTs (*N* = 39)

Heading/subheading	Criterion	Yes (%)	95% confidence interval (%)
Title and abstract	*Phrase ‘step wedge’ or ‘stepped wedge’ used*	*26 (66.7)*	*(51.0, 79.4)*
	**Phrase ‘randomized’ used**	**29 (74.4)**	**(58.9, 85.4)**
Introduction or methods	*Rationale for stepped wedge design given*	*25 (64.1)*	*(48.4, 77.3)*
	**Rationale for clustering given**	**6 (15.4)**	**(7.2, 29.7)**
	**Specific objectives of the trial given**	**39 (100.0)**	**(91.0, 100.0)**
	*Diagram of the design provided*	*26 (66.7)*	*(51.0, 79.4)*
	**Description of the trial design provided**	**36 (92.3)**	***(79.7, 97.3)***
	**Eligibility criteria for clusters provided**	**25 (64.1)**	**(48.4, 77.3)**
	**Eligibility criteria for individuals provided**	**34 (87.2)**	**(73.3, 94.4)**
	**Settings and locations where data collected adequately described**	**39 (100.0)**	**(91.0, 100.0)**
	**Interventions adequately described**	**37 (94.9)**	**(83.1, 98.6)**
	**Completely defined primary outcomes**	**36 (92.3)**	**(29.3, 59.0)**
	Completely defined secondary outcomes*	**22 (95.7)**	**(79.0, 99.2)**
	***Justification for sample size provided***	***24 (61.5)***	***(45.9, 75.1)***
	**Use or non-use of intracluster correlation coefficient or coefficient of variation stated**	**17 (43.6)**	**(29.3, 59.0)**
	Type-I error rate used stated	22 (56.4)	(41.0, 70.7)
	Type-II error rate for design stated	23 (59.0)	(43.4, 72.9)
	**Method of random allocation used**	**23 (59.0)**	**(43.4, 72.9)**
	**Type or randomization used**	**36 (92.3)**	**(29.3, 59.0)**
	**Allocation concealment mechanism used**	**12 (30.8)**	**(18.6, 46.4)**
	**Who implemented the randomization detailed**	**15 (38.5)**	**(24.9, 54.1)**
	**Consent sought from**	**31 (79.5)**	**(64.5, 89.2)**
	**Blinding adequately described**	**20 (51.3)**	**(36.2, 66.1)**
Results	Flow diagram provided	24 (61.5)	(45.9, 75.1)
	**Losses and exclusions detailed**	**30 (76.9)**	**(61.7, 87.4)**
	**Dates of the trial provided**	**33 (84.6)**	**(70.3, 92.8)**
	*Dates of each time period provided*	*14 (35.9)*	*(22.7, 51.6)*
	**Baseline data reported**	**33 (84.6)**	**(70.3, 92.8)**
	***Final number of clusters analyzed detailed***	***39 (100.0)***	***(91.0, 100.0)***
	***Final number of individuals analyzed detailed***	***36 (92.3)***	***(29.3, 59.0)***
	*Final number of steps detailed*	*38 (97.4)*	*(86.8, 99.5)*
	**Summary of outcomes provided**	**39 (100.0)**	**(91.0, 100.0)**
	***Point estimate and variation estimate of primary outcome measures provided***	***32 (82.1)***	***(67.3, 91.0)***
	Point estimate and variation estimate of secondary outcome measures provided*	**15 (65.2)**	**(44.9, 81.2)**
	Intention-to-treat analysis used	16 (41.0)	*(27.1, 56.6)*
	***Intracluster correlation coefficient or coefficient of variation value reported***	***6 (15.4)***	***(7.2, 29.7)***
Discussion	**Potential harms detailed**	**6 (15.4)**	**(7.2, 29.7)**
	**Generalizability of results described**	**18 (46.2)**	**(31.6, 61.4)**
	**Limitations of the trial described**	**38 (97.4)**	**(86.8, 99.5)**
	**Interpretation of the results provided**	**39 (100.0)**	**(91.0, 100.0)**
	**Trial registration provided or referenced**	**17 (43.6)**	**(29.3, 59.0)**
	**Trial protocol provided or referenced**	**14 (35.9)**	**(22.7, 51.6)**
	**Trial funding detailed**	**37 (94.9)**	**(83.1, 98.6)**
Overall median		– (66.7)	
**Boldface median (CONSORT)**		**– (80.8)**	
*Italicized median (key)*		*26 (66.7)*	

Indicators listed in bold are those contained in the CONSORT extension to CRCTs. Indicators listed in italics are those contained in the chosen subset of 10 key criteria. The two criteria marked with a * are out of 25 records rather than 39, since secondary outcomes were not present in every trial. An overall median raw mark, and median raw mark on the CONSORT criteria are consequently not calculable

We observe that the percentage of the trial reports meeting each criteria varied from 15.4% to 100.0%, with an overall median of 66.7%. The median performance across the 10 key criteria was also 66.7%; however, it was 80.8% for those criteria listed on the CONSORT extension to CRCTs. Note that the Pearson correlation between the individual trial performance on the overall and CONSORT criteria was 0.97, between the overall and key criteria was 0.80, and between the CONSORT and key criteria was 0.69, to two decimal places.

Highlighting several figures, we see very few studies that detailed why clustered allocation was used in their trial (6/39, 15.4%). 35.9% (14/39) did not justify use of a SW-CRCT design, and only 61.5% (24/39) provided a flow diagram of any kind. 41.0% (16/39) of the studies used an intention-to-treat analysis, and 48.7% (19/39) of the studies did not adequately detail who was blinded to the intervention.

From Fig. 4a we see that the quality of reporting of each individual trial is generally poor, with one study meeting 39.0% of the criteria. However, from the Loess smoother [47], and its 95% confidence interval, there does appear to be somewhat of an upward trend in the reports’ percentage scores as we approach the present day. Similar statements hold when assessing the criteria listed on the CONSORT extension to CRCTs (Fig. 4b), and also the 10 key criteria (Fig. 4c). Nonetheless, there were, worryingly, still four studies published in either 2014 or 2015 that met fewer than 60% of the CONSORT criteria, and one study in 2014 meeting 40% of the key criteria.

### Primary outcome measure results

Our analysis of the full trial reports utilizing SW-CRCT designs revealed that when a minimum clinically important difference had been specified for a primary outcome measure, or measures, 52.0% (13/25) of these studies did not find a significant effect of the intervention. This is perhaps surprising, given the aforementioned notion that SW-CRCTs are commonly used when there is an *a-priori* belief that the intervention will be effective. In contrast, however, when no minimum clinically important difference was specified for a primary outcome measure, or measures, 85.0% (17/20) of studies found a significant effect.

## Discussion

The SW-CRCT design has received substantial attention over the last few years. In this work, we have addressed some of the issues associated with it through a literature review. Principally, we sought to discern the reporting quality of completed SW-CRCTs. To this end, we presented a review of 39 completed studies, assessing their reporting quality through 43 chosen indicators. Previously conducted reviews on SW-CRCTs have assessed reporting quality on at most 15 completed studies and 23 indicators at once. Therefore, we here provided analysis based on a far larger number of studies and indicators than previous reviews.

Our analysis found that much work remains to be done to improve quality. Sound reporting is key to assessing the validity of a study; however, the median performance across all considered criteria was 66.7%. Moreover, whilst the type of randomization used was well described (37/39, 92.3%), the method of random allocation (23/39, 59.0%), the allocation concealment mechanism (12/29, 30.8%) and the person who implemented the randomization (15/39, 38.5%) was not. Moreover, 25.6% (10/39) of studies did not describe their designs as randomized in the title or abstract.

Some 15.4% (6/39) of studies successfully detailed any harm associated with their intervention. In many instances this may well be because there was no harm, but it is important to make this clear, particularly as many of the interventions involve drugs for which side-effects could well be expected.

In line with a 2016 review [35], 38.5% (15/39) of studies did not provide any form of justification for their sample size, and 43.6% (17/39) mentioned the intracluster correlation or coefficient of variation. Moreover, 43.6% (17/39) and 41.0% (16/39) of the studies did not detail planned type-I and type-II error rates for their design. Thus, again there is great room for improvement in the reporting of the specification of SW-CRCTs.

Nonetheless, from Fig. 4 it does appear somewhat that the standard of reporting of SW-CRCTs has improved since 2010. This improvement may be because more has now been published about the design, or could perhaps be a result of the lag time for journals adopting the new criteria contained within the CONSORT extension to CRCTs [37, 38].

Whilst the median performance across the criteria listed on the CONSORT extension to CRCTs was 80.8%, it is more alarming that the mean performance was 70.8%. This, however, should perhaps not surprise us, as it is not a problem restricted solely to SW-CRCTs. Indeed several recent reviews have identified that the reporting of CRCTs in several areas has been poor [48–51]. In particular, one 2011 review identified that the quality of reporting of only 5 of 14 considered criteria had improved following the publication of the CONSORT extension to CRCTs [51]. However, this may have been the result of a lag of uptake of the new criteria. Regardless, further efforts are needed to improve reporting quality for not only SW-CRCTs, but all CRCTs. Nonetheless, there are several features specific to SW-CRCTs not contained within this CONSORT extension, and therefore further guidance is needed on publication of SW-CRCTs. Fortunately, therefore, a CONSORT extension to SW-CRCTs is now under development [52]. In this extension, it appears warranted to encourage greater detail on the timing of each step, to ensure that it is improved in the future. It will also, in light of recent extensions to the classical SW-CRCT, be important to define clearly what constitutes a SW-CRCT, and therefore which trials would need to adhere to such guidance. Finally, 33.3% (13/39) of trials completed thus far did not include a diagram of their design. This is strongly encouraged by the CONSORT extension to CRCTs. However, it may be wise for it to be listed as a specific point on an extension to SW-CRCTs, perhaps even with certain required features, as it is clearly a very powerful means of detailing a SW-CRCT design.

In addition, we sought to ascertain the general design features of all SW-CRCTs conducted to date. To this end, we presented a review of features from an additional 84 study protocols, registrations and conference reports, along with those from the aforementioned 39 completed studies.

We observe that 91% of the identified and included records were from 2010 onwards. Thus, it appears that the design is increasing in popularity, perhaps reflecting an increasing acknowledgement of its usefulness, or simply that more is now known about the design.

It is clear that the design has been used in an extremely broad range of research areas, in a wide range of countries. Moreover, the actual characteristics of the design have varied substantially as well. Therefore, it seems that the design has proven useful to a large selection of study scenarios, its benefits seemingly coming from reasons other than one specific trial scenario. Indeed, our work highlighted once again some of the advantages of the SW-CRCT design that have frequently been cited.

We identified that the most common reason for use was that trial organizers wished to give the intervention to all clusters eventually. Additionally, a large number cited logistical and practical constraints that made the staggered implementation involved in a SW-CRCT favourable. A small number of studies cited that the design was preferred for efficiency or power related reasons. It is, of course, essential to remember that this is not universally true of the SW-CRCT design. Moreover, some studies cited that the design would allow them to fine-tune the intervention over time, but it is important to note that in SW-CRCTs one should not set out to change the intervention across time periods as this would imply that the intervention effect would change during the trial. Overall, however, it seems that there are a large number of reasons for which the design has been preferred, contributing to the broad array of research domains in which it has been utilized.

There are also several disadvantages associated with the SW-CRCT design [27, 29, 31]. Specifically, if the chosen SW-CRCT design is one that requires data collection at each point when a new cluster receives the experimental intervention, this could, in some circumstances, see the cost of data collection become considerable. Given that the design is also not guaranteed to require a smaller sample size than a parallel group CRCT, it may also require a substantially longer trial duration. Consequently, as discussed recently, researchers must remain careful when deciding whether to employ the design [29]. This seems particularly true in light of our findings that 52.0% of completed studies did not find a significant effect on any primary outcome measure for which a minimum clinically important difference had been specified. Of course, it could be that these studies were simply underpowered, owing to misspecification of the variance parameters. Extension of the classical SW-CRCT design to incorporate sample size re-estimation could therefore be an important advance. Alternatively, to guard against over-enthusiastic use of the design, interim analyses to stop the trial early for futility may also be a useful design extension for trial organizers to consider. This approach, however, would, of course, not always be applicable; for example if the intervention were part of some wider planned roll out, or if the possibility that some clusters might not receive the intervention was deemed unacceptable.

### Strengths and limitations

We used a literature search strategy based on a previously completed review to identify relevant articles for inclusion. In particular, we utilized a large number of search terms, across a large number of databases, and set no limits on the publication date. Therefore, we were able to complete an assessment of the general design features and quality of reporting of SW-CRCTs to date on more trials than all previous reviews. However, it is still possible that some studies might have been missed that used other phrases to describe their design that we did not include in our search. Additionally, we might have missed some studies reporting in languages other than English. Finally, several reviews, ours included, have now looked to include SW-CRCTs conducted in non-health based research settings. However, to maximize the chance of such studies being identified, additional databases should be included, such as the Campbell Collaboration [53].

We chose to exclude studies where there was no (baseline) period present in which no clusters received the experimental intervention; however, following recent publications many might consider designs of this form as SW-CRCTs. Moreover, similar statements are true for incomplete block SW-CRCTs. Future researchers might seek to include such studies in their reviews. Furthermore, we chose to assess reporting quality on a particular set of 10 criteria, which we chose to be key. Whilst justification for our choices was discussed, some researchers might have preferred alternate criteria to be included in this list. It is possible that future work could convene a panel of experts on SW-CRCT designs to determine which criteria to view as of paramount importance, enlisting the help, for example, of the Comet Initiative [54] or the Equator Network [55].

In addition, future reviews could seek to expand the classification of their extracted data beyond our, and the previous reviewers’, simple ‘yes or no’ prescription. For some of the considered criteria, it would be particularly beneficial to incorporate whether they were partially satisfied, according to some designated scoring procedure. This would potentially allow the improvement of the reporting of SW-CRCTs to be more accurately measured.

Finally, only one author conducted the inclusion search and the eventual data extraction. In our initial planning of the review, we did consider the use of duplicate abstract screening and data extraction. However, it was decided one author would perform all of the screening and data extraction, marking the cases where decisions were unclear for joint discussion with the other authors. This was because, in nearly all cases, it was very clear what decision should be made, with this fact assisted by the careful choice of criteria that avoided the need for any subjective opinion. We acknowledge that this deviates from best practice for conducting a review but are confident it has not affected the quality of our work. For record selection, this claim is backed up by the verification that all trials included in previous reviews, that met our choice of inclusion criteria, were included in our review.

## Conclusions

We identified that regardless of evidence of recent improvements, the quality of reporting of SW-CRCTs has been low. There is therefore a need for further guidance on how to report such trials, and the CONSORT extension to SW-CRCTs, currently under development, certainly seems warranted. Through this, precisely which information should be included when presenting the results of a SW-CRCT, and what constitutes a SW-CRCT, should be clarified, such that future SW-CRCTs may be better reported.

## Additional files

Additional file 1: Review methodology. (DOCX 50 kb)
Additional file 2: PRISMA checklist. (DOCX 31 kb)
Additional file 3: Included studies and the information extracted from them, along with the records from each of the major stages of the review. (XLSX 5750 kb)

## Abbreviations

CRCT: Cluster randomized controlled trial
RCT: Randomized controlled trial
SW: Stepped wedge
