# Supplementary material for: Stepped wedge cluster randomized controlled trial designs: a review of reporting quality and design features
Source: Trials. 2017 Jan 21;18:33. doi: 10.1186/s13063-017-1783-0 (PMC5251280; doi:10.1186/s13063-017-1783-0)
Supplement: Additional file 1: — Review methodology. (DOCX 50 kb) [file 13063_2017_1783_MOESM1_ESM.docx]

**Review methodology**

**Protocol**

**Introduction**

Five major reviews on the use of the stepped wedge (SW) trial design have been completed to date [1-5]. The first resolved that the design affords several advantages and advocated its use accordingly in appropriate scenarios [1]. This review included both randomised and non-randomised studies, as well as those with individual and cluster level allocation, limiting its search to the health sector. In total it included 12 studies. Mdege *et al* (2011) expanded their search to include non-health sector studies, but focused on randomised trials with cluster allocation [3]. They included 25 trials in their review, and gave significant attention to the reporting standards of the published SW studies. Beard *et al* (2015) searched for SW randomised trials, with individual or cluster level allocation, published between 2010 and 2014 [3]. They included 37 trials in their analysis, focusing their attention upon the qualitative features of the studies and the methods used to calculate sample sizes and analyse collected data. Martin *et al* (2016) searched for SW randomised trials with cluster level allocation, assessing adherence to each of the 9 sample size calculation items recommended in the 2012 CONSORT extension for cluster randomised controlled trials (CRCTs) [4]. Finally, Barker *et al* (2016) [5] conducted a review of the literature of the statistical methodology used in SW-CRCTs.

Here, we complete the most up-to-date analysis on the characteristics of SW designs, reasons investigators have employed the SW design, and importantly critically assess the reporting standards of completed studies across the CONSORT extension for CRCTs [6,7]. We limit ourselves to randomised studies with cluster level allocation (henceforth referred to as SW-CRCTs), but do not limit the subject area in which the study was performed. Furthermore, we give additional focus to the percentage of completed studies which did not find a significant effect on any of their stated primary outcome measures.

**Aim**

To establish the degree to which the SW-CRCT design has been utilised.

**Objectives**

- To determine the areas in which the SW-CRCT design has been used.
- To determine the general characteristic design features of SW-CRCTs.
- To determine the motivations for employing the SW-CRCT design.
- To determine the methods of analysis that have been used with SW-CRCT data.
- To determine the quality of reporting of published SW-CRCTs.
- To determine the percentage of such published SW-CRCTs which did not find a significant effect of the intervention upon any of the primary outcomes measures.

**Methods**

A completed PRISMA checklist is available as Additional file 3 [8].

**Database search**

The following electronic databases will be searched: Pubmed (incorporating Medline), Ovid (incorporating Embase), PsycINFO, Cochrane Library, Web of Knowledge, the ISRCTN registry and ClinicalTrials.gov.

The following search phrases will be used: stepped wedge, step wedge, experimentally staged introduction, delayed intervention, one directional cross over design and the fifteen possible combinations of incremental/phased/staggered/step wise/delayed and recruitment/introduction/implementation. Further information on how these search terms were entered is listed below.

In addition, articles will also be identified from the reference lists of the included studies, as well as the list of citations for these included studies according to Google Scholar. Moreover, the citations of the three most cited papers relating to SW-CRCTs (according to a search for “stepped wedge” on Google Scholar) will also be used [1,2,9].

The search will be limited to articles published in English only, and no publication date limits will be applied.

**Study inclusion and exclusion criteria**

We define a SW-CRCT as follows (with *and* here requiring both the preceding and succeeding criteria to be met):

1. Cluster level allocation was utilised to compare experimental interventions with controls;
2. data were gathered in at least three time periods on at least two clusters;
3. each cluster begins in a control condition and changes from this control to an experimental intervention exactly once during the study, and then remains on this experimental intervention;
4. the time point at which clusters change from control states to experimental ones is randomised, and not identical for all clusters;
5. data are gathered for every included cluster in every time period; and
6. an analysis is performed and reported on at least one primary outcome measure, for each experimental intervention, incorporating data from a time period in which all clusters received the intervention.

Inclusion criteria are then:

- Reports, protocols, trial registrations and conference presentations on original research studies that used/plan to use the SW-CRCT design, from all fields of research.
- English language.

Specifically, this leaves the following as key exclusion criteria (as well as the obvious criterion of not discussing a SW-CRCT):

- Studies retrospectively analysed as a SW-CRCTs when not originally designed as such.
- Reports, protocols, trials registrations and conference presentations on original research studies for which a full report has been published.

**Selection of studies for inclusion in the review**

MJG will read all titles/and or abstracts retrieved from the database search process and eliminate obviously irrelevant records. The full text of the remaining possibly relevant studies will then be obtained and read, with each record classified as clearly relevant (meets all inclusion criteria), clearly irrelevant, or insufficient information to make a decision. For those cases with insufficient information, corresponding author(s) will be contacted for further information to make a final decision on relevance. In the instance that the full text of a possibly relevant study cannot be retrieved, or corresponding author(s) are contacted and no reply is received, the record will be excluded from the final review.

To qualitatively examine the performance of our selection process, we will then compare the included studies from previous reviews to ours.

**Data extraction and management**

MJG will extract data from all included records according to a set of pre-chosen questions (see Appendix). This information will include study identifiers, information on the area of research, motivations for employing the SW-CRCT design, general characteristics of the SW-CRCT design, and the method of data analysis. Additionally, for completed trial reports data will be extracted on numerous questions assessing quality of reporting. All data will be stored in an Excel spreadsheet (Additional file 2). The questions will be piloted on 10 papers and adjustments made where appropriate. Where conclusions are unclear, final decisions were made through discussion with additional authors (JMSW and APM).

**Reporting quality assessment**

The quality of reporting to date will be assessed through performance on the 43 criteria listed in the Appendix. In addition, performance on the 34 of these criteria listed on the CONSORT extension to CRCTs [6] will be assessed, since this provides the best available guidance at present on the publication of SW-CRCTs. Finally, a set of ten ‘key’ criteria have been chosen to assess performance. These criteria were a group decision between the authors, and are specifically a small set that would not only allow the trial to be identified more quickly as employing a SW-CRCT design, but also would allow the results of the trial to be used more easily in the future development of new methodology. To ease the identification of the design as a SW-CRCT we include

- *Phrase “step wedge” or “stepped wedge” used;*
- *Rationale for stepped wedge design given;*
- *Diagram of the design provided.*

Then to allow each of the trials main design features to be determined we include

- *Dates of each time period provided;*
- *Final number of clusters analysed detailed;*
- *Final number of individuals analysed detailed;*
- *Final number of steps detailed;*

Finally, to ease the use of the trial as a motivating example in future methodological research we include

- *Justification for sample size provided;*
- *Point estimate and variation estimate of primary outcome measures provided;*
- *ICC/CV value reported.*

In particular, these would in many cases allow the efficiency of alternate designs to be compared to that employed. As has been noted previously reporting of the ICC/CV is especially important to this [10], and has been perhaps badly reported in the past.

**Search Strategy**

All searches were performed on the 24^th^ February 2015. For each database, each of the 20 search terms was independently searched for and the resulting records saved to file. Following this, for each database in turn, identified records were merged into one .csv file using R v. 3.1.3 [11]. Duplicate records were then removed where possible using text string matching, and then further removed by hand. This resulted in seven .csv files containing final lists of records identified from each database. These .csv files were then merged in to one, and by hand de-duplicated to give a final .csv file containing all unique identified records from online database searching. Separately, an Excel file was continuously updated to store any additional records identified by other means. The records in these two Excel files were then screened for inclusion according to the methodology discussed above. Further details on the terms entered to each database are given below.

**Pubmed**

In the Advanced Search Builder, ‘All Fields’ was in turn set to each of the 20 search terms e.g. “stepped wedge”. Results were downloaded as a .txt file.

**Ovid**

Embase 1974 to 2015 Week 6 was included. In the Advanced Search tab, in turn each of the 20 search terms were entered as a ‘Keyword’ e.g. “stepped wedge”. Results were downloaded as a .txt file.

**PsycINFO**

In the Advanced Search tab, ‘Any Field’ was in turn set to each of the 20 search terms e.g. “stepped wedge”. Results were downloaded as a .ris file.

**Cochrane Library**

In the Search tab, ‘Search All Text’ was in turn set to each of the 20 search terms e.g. “stepped wedge”. Results were downloaded as a .txt file.

**Web of Knowledge**

In a Basic Search, ‘Topic’ was in turn set to each of the 20 search terms e.g. “stepped wedge”. ‘TIMESPAN’ was set to ‘All years’. Results were downloaded as a .txt file.

**ISRCTN registry**

In an Advanced Search, ‘Text search’ was in turn set to each of the 20 search terms e.g. “stepped wedge”. Results were copied by hand (title of study, registry number, primary contact) in to a .csv file.

**ClinicalTrials.gov**

In an Advanced Search, ‘Search Terms’ was in turn set to each of the 20 search terms e.g. “stepped wedge”. Results were downloaded as a .csv file.

**Results**

10 773 records were identified through database searching. The final record numbers for the seven databases after the removal of duplicates from each (caused by searching for each term independently) were as follows:

| Database | Number of records |
| --- | --- |
| Pubmed | 2260 |
| Ovid | 4937 |
| PsycINFO | 181 |
| Cochrane Library | 97 |
| Web of Knowledge | 1839 |
| ISRCTN registry | 49 |
| ClinicalTrials.gov | 167 |
| Total | **9530** |

Additionally, 51 records were identified from other sources. The final number of unique records once these results were merged and de-duplicated was 7720. Of these 472 records were included for full text analysis to determine possible final inclusion. Of these, 123 were eligible (39 completed trial reports, 84 trial protocols/registrations/conference presentations), and underwent data extraction. The results of this data extraction is presented in the main article. Figures on the first identified reason for exclusion following full text analysis are provided in Figure 1. These are broken down into ten categories, the details of which are as follows

- **PG-CRCT:** Record found to be related to a PG-CRCT, rather than a SW-CRCT.
- **Superseded by another record:** Record found to be for example the protocol of a now completed trial with published results, or the registration of a trial for which there is now a published protocol available.
- **Individual level randomisation:** Record found to be related to a trial with individual level, rather than cluster, allocation.
- **Non-randomised allocation:** Record found to be related to a trial with non-randomised allocation to the intervention.
- **Preliminary/Secondary publication:** Record found to be a preliminary or secondary publication related to an included record.
- **Discusses SW designs:** Record found to simply discuss SW-CRCT designs, and not present a trial using the design.
- **Phased implementation of an intervention:** Record pertained to a trial where multiple components of an intervention where phased in over time.
- **No response:** No response to contact attempting to clarify the utilised design.
- **Incomplete block:** Record found to be related to an incomplete block designed trial, breaking our requirement for data to be gathered on each included cluster, in each time period.
- **Other:** Other reason for exclusion; such as trial retrospectively analysed as a SW-CRCT, or trial utilised some other study design.

**References**

1. Brown CA, Lilford RJ. The stepped wedge trial design: a systematic review. BMC Med Res Methodol 2006; 6:54.
2. Mdege N, Man M, Taylor (nee Brown) CA, Torgerson DJ. Systematic review of stepped wedge cluster randomized trials shows that design is particularly used to evaluate interventions during routing implementation. J Clin Epidemiol 2011; 64:936-948.
3. Beard E, Lewis JJ, Copas A, Davey C, Osrin D, Baio G, Thompson JA, Fielding KL, Omar RZ, Ononge S, Hargreaves J, Prost A. Stepped wedge randomised controlled trials: systematic review of studies published between 2010 and 2014. Trials 2015; 16:353.
4. Martin J, Taljaard M, Girling A, Hemming K. Systematic review finds major deficiencies in sample size methodology and reporting for stepped-wedge cluster randomised trials. BMJ Open 2016; 6:e010166.
5. Barker D, McElduff P, D’Este C, Campbell MJ. Stepped wedge cluster randomised trials: a review of the statistical methodology used and available. BMC Med Res Methodol 2016; 16:69.
6. Campbell MK, Elbourne DR, Altman DG; for the CONSORT Group. CONSORT statement: extension to cluster randomised trials. BMJ. 2004;328(7441):702-708.
7. Campbell MK, Elbourne DR, Altman DG; for the CONSORT Group. CONSORT 2010 statement: extension to cluster randomised trials. BMJ. 2012;345:e5661.
8. Moher D, Liberati A, Tetzlaff J, Altman DG, The PRISMA Group. Preferred reporting items for systematic reviews and meta-analyses: The PRISMA statement. PloS Med. 2009;6(7):e1000097.
9. Hussey MA, Hughes JP. Design and analysis of stepped wedge cluster randomised trials. Contemp Clin Trials 2007; 28:182-191.
10. Campbell MK, Grimshaw JM, Elbourne DR. Intracluster correlation coefficients in cluster randomized trials: empirical insights into how should they be reported. BMC Med Res Methodol 2004; 6:9.
11. R Core Team. R: A Language and Environment for Statistical Computing. Vienna, Austria. 2016; http://www.r-project.org/.

**Appendix**

The following information was extracted from all included studies:

| Characteristic | Example |
| --- | --- |
| Type of record | Report |
| Lead author | Smith |
| Title | A stepped wedge cluster randomised trial to evaluate… |
| Publication year | 2014 |
| Subject area | Health |
| Disease/Domain | Cancer |
| Nature of intervention | Vaccination |
| Setting | Hospitals |
| Country | USA |
| Received industry support | Yes |
| Motivations for SW-CRCT design use | Ethical |
| Level of stepping | Wards |
| Number of steps | 10 |
| Number of clusters | 30 |
| Time period between steps | 2 months |
| Design type | Open cohort |
| Primary outcome measures | Incidence of cancer |
| Statistical method of data analysis | Linear mixed model |
| Intended sample size | 3300 |

The following further information was obtained from the completed trial reports in order to assess the quality of reporting of SW-CRCTs. These criteria were based upon those employed previously [2], and the CONSORT extension for CRCTs [6,7], with each assessed to be either ‘Y’ (for Yes) or ‘N’ (for No). The italicised criteria indicate those forming the subset of ‘key’ criteria, whilst those in bold are listed on the CONSORT extension to CRCTs [6,7]:

| Heading/subheading | Criterion |
| --- | --- |
| Title and abstract | *Phrase “step wedge” or “stepped wedge” used* |
|  | **Phrase “randomised” used** |
| Introduction/Methods | *Rationale for stepped wedge design given* |
|  | **Rationale for clustering given** |
|  | **Specific objectives of the trial given** |
|  | *Diagram of the design provided* |
|  | **Description of the trial design provided** |
|  | **Eligibility criteria for clusters provided** |
|  | **Eligibility criteria for individuals provided** |
|  | **Settings and locations where data collected adequately described** |
|  | **Interventions adequately described** |
|  | **Completely defined primary and secondary outcomes** |
|  | **Completely defined secondary outcomes** |
|  | ***Justification for sample size provided*** |
|  | **Use or non-use of ICC/CV stated** |
|  | Type-I error rate used stated |
|  | Type-II error rate for design stated |
|  | **Method of random allocation used** |
|  | **Type or randomisation used** |
|  | **Allocation concealment mechanism used** |
|  | **Who implemented the randomisation detailed** |
|  | **Consent sought from** |
|  | **Blinding adequately described** |
| Results | Flow diagram provided |
|  | **Losses and exclusions detailed** |
|  | **Dates of the trial provided** |
|  | *Dates of each time period provided* |
|  | **Baseline data reported** |
|  | ***Final number of clusters analysed detailed*** |
|  | ***Final number of individuals analysed detailed*** |
|  | *Final number of steps detailed* |
|  | **Summary of outcomes provided** |
|  | ***Point estimate and variation estimate of primary outcome measures provided*** |
|  | **Point estimate and variation estimate of secondary outcome measures provided** |
|  | Intention-to-treat analysis used |
|  | ***ICC/CV value reported*** |
| Discussion | **Potential harms detailed** |
|  | **Generalisibility of results described** |
|  | **Limitations of the trial described** |
|  | **Interpretation of the results provided** |
|  | **Trial registration provided/referenced** |
|  | **Trial protocol provided/referenced** |
|  | **Trial funding detailed** |
